# Could a reduced hemoglobin, albumin, lymphocyte, and platelet (HALP) score predict autoimmune hepatitis and degree of liver fibrosis?

**DOI:** 10.1590/1806-9282.20230905

**Published:** 2024-01-26

**Authors:** Muge Ustaoglu, Gulali Aktas, Omer Kucukdemirci, Ibrahim Goren, Berk Bas

**Affiliations:** 1 Ondokuz Mayis University, Faculty of Medicine, Department of Gastroenterology – Samsun, Turkey.; 2 Abant Izzet Baysal University Hospital, Department of Internal Medicine – Bolu, Turkey.

**Keywords:** Autoimmune hepatitis, HALP score, Inflammation, APRI score, FIB-4 score, Fibrosis

## Abstract

**OBJECTIVE:**

Autoimmune hepatitis is a rare inflammatory disease of the liver that is characterized by elevated liver enzymes. The hemoglobin, albumin, lymphocyte, and platelet score, which is derived from hemoglobin, serum albumin, circulating lymphocyte count, and platelet count, is also associated with inflammatory conditions. The aim was to examine the hemoglobin, albumin, lymphocyte, and platelet score of patients with autoimmune hepatitis and to compare it to that of healthy individuals in this retrospective analysis.

**METHODS:**

Subjects diagnosed with autoimmune hepatitis were enrolled in the study, and healthy individuals were enrolled as controls. Moreover, autoimmune hepatitis subjects were grouped into mild or moderate/advanced fibrosis. Furthermore, aspartate to platelet ratio index, Fibrosis-4, and hemoglobin, albumin, lymphocyte, and platelet scores of the autoimmune hepatitis patients and controls were compared. In addition, the hemoglobin, albumin, lymphocyte, and platelet score of the autoimmune hepatitis patients with mild fibrosis is compared to that of those with moderate/advanced fibrosis.

**RESULTS:**

The mean hemoglobin, albumin, lymphocyte, and platelet score of the autoimmune hepatitis patients was 44.2±14.5 while this value was 76.8±15.5 in control subjects. The hemoglobin, albumin, lymphocyte, and platelet score was significantly reduced in autoimmune hepatitis patients than healthy controls (p<0.001). The hemoglobin, albumin, lymphocyte, and platelet score was significantly and negatively correlated with C-reactive protein, aspartate, alanine transaminase, gamma glutamyl transferase, aspartate to platelet ratio index, and Fibrosis-4 values. A hemoglobin, albumin, lymphocyte, and platelet score that was lower than 52.3 had 83% sensitivity and 73% specificity in predicting autoimmune hepatitis. The sensitivity and specificity of the hemoglobin, albumin, lymphocyte, and platelet score were higher than the Fibrosis-4 score in predicting moderate/advanced fibrosis in autoimmune hepatitis.

**CONCLUSION:**

We suggest that the hemoglobin, albumin, lymphocyte, and platelet score be used as an additional noninvasive diagnostic tool for autoimmune hepatitis and to predict moderate/advanced liver fibrosis in patients with autoimmune hepatitis.

## INTRODUCTION

Autoimmune hepatitis (AIH) is a rare liver condition characterized by elevated liver enzymes and positive specific antibodies in the serum of the affected subjects^1^. An increased burden of inflammation is a hallmark feature of autoimmune hepatitis^2,3^ and other liver diseases^4^.

Establishing the diagnosis of AIH is challenging. Therefore, novel markers are studied in this population to make a concise diagnosis and also to predict the outcome^5^. One of these novel markers is the hemoglobin, albumin, lymphocyte, and platelet (HALP) score. The association between inflammatory conditions, such as heart failure^6^, intestinal obstruction^7^, stroke^8^, cancer^9^, and HALP score, has been well established in the medical literature. However, there are no published works about the role of the HALP score in subjects with AIH.

We aimed to examine the HALP score of the patients with AIH and compare it to that of healthy individuals in this retrospective analysis.

## METHODS

### Design, setting, and population

Subjects diagnosed with AIH in outpatient gastroenterology clinics at Ondokuz Mayis University Hospital between August 2020 and December 2022 were enrolled in the study after obtaining ethical approval from the local ethics committee (approval no. 2022/510). Control subjects were healthy individuals who presented to the internal medicine outpatient clinics of the institution for routine control. The exclusion criteria were as follows: younger than 18 years of age, active infectious or other inflammatory diseases, pregnancy, hematological conditions that alter platelet or lymphocyte counts, and any type of malignant condition. We also excluded subjects with advanced heart failure, chronic kidney disease, and chronic obstructive pulmonary disease.

Age, gender, and laboratory characteristics, such as serum biochemistry (urea, creatinine, aspartate transaminase [AST], alanine transaminase [ALT], albumin, C-reactive protein [CRP], alkaline phosphatase [ALP], and gamma glutamyl transferase [GGT]); autoimmune markers (anti-nuclear antibody [ANA], anti-mitochondrial antibody [AMA], anti-smooth muscle antibody [ASMA], and anti-liver kidney antibody 1 [anti-LKM1]); and hemogram parameters (white blood cell [WBC] count, neutrophil [neu] count, lymphocyte [lym] count, Hb, hematocrit [Htc], and platelet [PLT] count) were evaluated. Markers of common hepatitis viruses were recorded from patients’ records. We also recorded liver biopsy findings, including histological activity index (HAI) score and fibrosis degree, in subjects with AIH.

We calculated the HALP score with the following formula: (Hb×serum albumin×lym)/PLT. AST to PLT ratio index (APRI) and Fibrosis-4 (FIB-4) scores were also calculated with the formulas AST/PLT and (age×AST)/(PLT×root square [ALT]), respectively. Data from the AIH and control groups were compared. We further grouped AIH patients into two: mild fibrosis group (fibrosis score: 0 or 1) and the moderate/advanced fibrosis group (fibrosis score: 2–6). Data from the mild and moderate/advanced fibrosis groups were also compared.

### Statistical analyses

Statistical analyses were conducted with the SPSS software (SPSS 16 for Windows, IBM Co., Chicago, IL, USA). The homogeneity of the variables was analyzed with the Kolmogorov-Smirnov test. The comparison of the variables with a homogeneous distribution was done with an independent sample t-test. These variables were expressed as mean±standard deviation (SD). The variables without a homogeneous distribution were compared with the Mann-Whitney U test and expressed as medians (min–max). The comparison of categorical variables was done with a chi-square test and given in numbers and percentages. Correlation between study variables was conducted with Pearson's correlation test or Spearman's correlation test, where appropriate. The sensitivity and specificity of the HALP score in predicting autoimmune hepatitis were analyzed with the receiver operating characteristic (ROC) curve analysis test. When the p-value is lower than 5%, it is considered statistically significant.

## RESULTS

The study population consisted of 204 subjects, with 112 in the AIH group and 92 in the control group. The mean ages of the AIH and control groups were 43.7±7.7 years and 41.1±9.8 years, respectively (p=0.17). Of note, 85 (76%) of the AIH group were women, while 64 (70%) of the control group consisted of female subjects. The genders of the groups were not statistically significant (p=0.31).

Serum creatinine (p=0.06), WBC (p=0.17), neu (p=0.054), and PLT (p=0.63) of the AIH and control groups were not statistically different. Serum albumin (p<0.001), Hb (p=0.001), and lym (p<0.001) levels of the AIH group were significantly lower than those of the controls. On the contrary, CRP (p<0.001), AST (p<0.001), ALT (p<0.001), GGT (p<0.001), and ALP (p<0.001) levels were significantly increased in AIH patients compared to the control subjects. Table 1 shows the data for the AIH and control groups.

**Table 1:** Data of the autoimmune hepatitis and control subjects.

		AIH	Control	p-value
Sex	Female [n, (%)]	85 (76)	64 (70)	0.31
	Male [n, (%)]	27 (24)	28 (30)	
		Mean±SD		
Age (years)		43.7±7.7	41.1±9.8	0.17
Albumin (g/dL)		4;04±0.64	4.45±0.29	<0.001
Hb (g/dL)		13±1.7	13.7±1.2	0.001
PLT (k/mm^3^)		232±61	227±43	0.63
HALP score		44.2±14.5	76.8±15.5	<0.001
		Median (min–max)		
WBC (k/mm^3^)		4.5 (2.4–21)	7 (4–10)	0.17
Neu (k/mm^3^)		3.4 (1.4–15.7)	3.2 (1.2–6.9)	0.054
Lym (k/mm^3^)		1.92 (0.6–8,8)	2.54 (1.1–4,8)	<0.001
Creatinine (mg/dL)		0.71 (0.37–1.58)	0.72 (0.67–0,92)	0.06
CRP (mg/L)		5.3 (0.3–162)	1.7 (0.2–3,6)	<0.001
AST (U/L)		124 (17–3785)	21 (13–43)	<0.001
ALT (U/L)		191 (10–3085)	28 (16–52)	<0.001
GGT (U/L)		114 (12–2861)	43 (33–61)	<0.001
ALP (U/L)		153 (23–1293)	67 (55–104)	<0.001
APRI		1.88 (0.13–41.9)	0.24 (0.11–0.53)	<0.001
FIB-4		2.63 (0.19–20.6)	0.72 (0.38–2.1)	<0.001

The median APRI scores of the AIH and control patients were 1.88 (0.13–41.9) and 0.24 (0.11–0.53), respectively (p<0.001). The median FIB-4 scores of the AIH and control groups were 2.63 (0.19–20.6) and 0.72 (0.38–2.1), respectively (p<0.001). The mean HALP score of the patients with AIH was 44.2±14.5, while this value was 76.8±15.5 in control subjects. The HALP score was significantly lower in AIH patients than in healthy controls (p<0.001). The HALP score was significantly and negatively correlated with CRP (r=-0.15, p=0.04), AST (r=-0.33, p<0.001), ALT (r=-0.34, p<0.001), GGT (r=-0.19, p=0.008), APRI (r=-0.28, p<0.001), and FIB-4 (r=-0.26, p<0.001) values.

**Figure 1:**
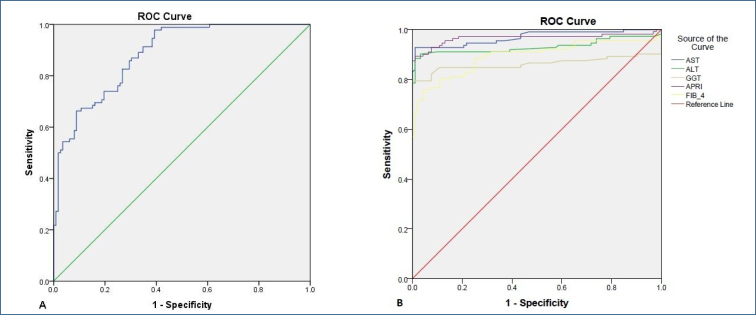
Receiver operating characteristic curve of hemoglobin, albumin, lymphocyte, and platelet score and other parameters in detecting autoimmune hepatitis.

The ROC analyses revealed that an HALP score lower than 52.3 has 83% sensitivity and 73% specificity in predicting AIH (AUC: 0.88, p<0.001, 95%CI 0.84–0.93). Figure 1(a) shows the ROC curve of the HALP score in detecting AIH.

In addition, the sensitivity and specificity of AST (when higher than 33.1 U/L) in predicting AIH were 93 and 99%, respectively (AUC: 0.97, p<0.001, 95%CI 0.95–0.992). The sensitivity and specificity of ALT (when higher than 40.5 U/L) in predicting AIH were 88 and 99%, respectively (AUC: 0.93, p<0.001, 95%CI 0.89–0.97). The sensitivity and specificity of GGT (when higher than 58.9 U/L) in predicting AIH were 80 and 93%, respectively (AUC: 0.86, p<0.001, 95%CI 0.80–0.92). The sensitivity and specificity of the APRI score (when higher than 0.35) in predicting AIH were 95 and 88%, respectively (AUC: 0.97, p<0.001, 95%CI 0.94–0.995). The sensitivity and specificity of the FIB-4 score (when higher than 0.99) in predicting AIH were 83 and 78%, respectively (AUC: 0.89, p<0.001, 95%CI 0.85–0.94). Figure 1(b) shows the ROC curves of these parameters for detecting AIH.

**Figure 2:**
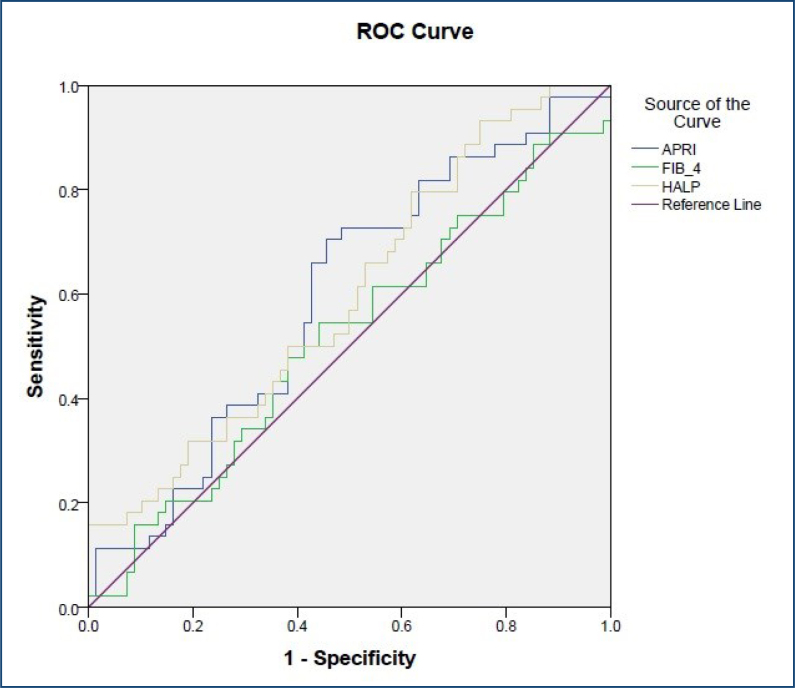
Receiver operating characteristic curves of the study variables in detecting moderate/advanced fibrosis.

Subgroup analysis was performed in the AIH group, which was divided into mild and moderate/advanced fibrosis groups. The HALP score was significantly lower in AIH patients with moderate/advanced fibrosis (41.7±4.1) compared to those with mild fibrosis (48.1±4.5) (p=0.02). Moreover, the HALP score was negatively and significantly correlated with the fibrosis degree in the AIH subgroup (r=0.4, p=0.002). However, an HALP score lower than 43.4 had 66% sensitivity and 47% specificity in detecting moderate/advanced fibrosis in AIH patients (AUC: 0.6, p=0.009, 95%CI 0.49–0.70). The sensitivity and specificity of the HALP score were higher than the FIB-4 score but lower than the APRI score in predicting moderate/advanced fibrosis in patients with AIH. Figure 2 shows the ROC curves of HALP, APRI, and FIB-4 scores in predicting moderate/advanced fibrosis in AIH patients.

## DISCUSSION

The present study showed that the HALP score could be used as an additional diagnostic tool in AIH since it is significantly reduced in these subjects compared to the controls. Another important outcome of our study is the significant and strong negative correlations between the HALP score and the levels of AST, ALT, GGT, APRI, and FIB-4 scores. Moreover, the HALP score was a useful predictor of advanced fibrosis in AIH subjects. Finally, the HALP score yielded considerably high sensitivity and specificity in establishing AIH diagnosis and predicting the degree of fibrosis in patients with AIH. A HALP score lower than 43.4 had 66% sensitivity and 47% specificity in detecting moderate/advanced fibrosis in AIH patients.

We shall speculate here why the HALP score is reduced in AIH. Inflammation is a hallmark feature of autoimmune liver diseases, including AIH^10^. Both portal and lobular inflammation are reported based on histopathological examination of the liver biopsies of the patients with AIH^11^. Involvement of even T cells in the inflammatory cites in AIH was reported by Longhi et al., 2 years ago^12^. Many reports suggest this phenomenon. For instance, a Chinese study revealed alterations in the gut microbiome may have beneficial effects on inflammation in an experimental AIH model^13^. Inflammatory markers have been suggested to be predictors of diagnosis and prognosis in AIH population^14^. These data suggest that inflammation is a characteristic of AIH. Indeed, in another study, nimbolide, an inflammatory agent, is shown to reduce inflammation in the AIH model in mice^15^. Moreover, steroids are the treatment of choice in AIH, which modulates inflammation.

Various reports in the literature concluded that the HALP score could be useful for diagnosing and predicting the prognosis of several conditions characterized by overt or subtle inflammation. Liver cancer has been reported to be associated with a reduced HALP score^16^. A decreased HALP score is not only seen in liver cancer but also in other gastrointestinal cancers^17^. It is also associated with stroke^18^, anti-neutrophil cytoplasmic antibody-associated vasculitis, and chronic obstructive pulmonary disease^19^. All of these conditions are associated with inflammation, as AIH is. Therefore, the association between AIH and the HALP score presented in this study is compatible with current literature.

The diagnosis of AIH is based on detectable autoantibodies, elevated to 1.1-folds of the upper limit of serum IgG, characteristic histological findings, and the absence of viral hepatitis^20^. We also established AIH based on this simplified criterion in the study population. Elevated aspartate and alanine transaminases, as well as GGT were common laboratory findings in patients with AIH. Similarly, we found elevated levels of these markers in patients with AIH compared to the control subjects, which is in line with literature data. Moreover, a significant and strong negative correlation between the HALP score and these enzymes was remarkable in the present study. Such a correlation between liver enzymes and red cell distribution width, a hemogram-based novel inflammatory predictor, has been proposed in a recent study^21^. Moreover, levels of CRP, a commonly used inflammatory marker, are correlated with liver enzymes and other markers of inflammation in AIH^22^. These data suggest that the correlation between liver enzymes and the HALP score presented in our study is consistent with literature data.

In one or two decades, APRI and FIB-4 scores have been introduced as prognostic markers of various liver diseases, from hepatitis to cirrhosis. Increased APRI and FIB-4 scores are suggested to be related to liver inflammation and fibrosis^23^. Moreover, elevated APRI and FIB-4 scores have also been reported in autoimmune liver diseases^3^. Similar to the literature findings, we reported increased APRI and FIB-4 scores in AIH patients compared to healthy controls. In addition, HALP score was also negatively correlated with these scores.

Several limitations are present in our study. Retrospective study design and a relatively small study population are two of these limitations. Another limitation can be stated as the single-centered nature of the study. However, to the best of our knowledge, this is the first study to find a significant association between the HALP score and AIH.

In conclusion, we suggest that the HALP score should be used by physicians as a noninvasive predictor in the diagnosis of disease in patients with suspected AIH and in predicting liver fibrosis in patients diagnosed with AIH.

## Data Availability

The data that support the findings of this study are available on reasonable request from the corresponding author. The data are not publicly available due to privacy or ethical restrictions.

## References

[1] Sucher E, Sucher R, Gradistanac T, Brandacher G, Schneeberger S, Berg T (2019). Autoimmune hepatitis-immunologically triggered liver pathogenesis-diagnostic and therapeutic strategies. J Immunol Res.

[2] Jiang R, Tang J, Zhang X, He Y, Yu Z, Chen S (2022). CCN1 promotes inflammation by inducing IL-6 production via α6β1/PI3K/Akt/NF-κB pathway in autoimmune hepatitis. Front Immunol.

[3] Ustaoglu M, Aktas G, Avcioglu U, Bas B, Bahceci BK (2021). Elevated platelet distribution width and red cell distribution width are associated with autoimmune liver diseases. Eur J Gastroenterol Hepatol.

[4] Kosekli MA, Kurtkulagii O, Kahveci G, Duman TT, Tel BMA, Bilgin S (2021). The association between serum uric acid to high density lipoprotein-cholesterol ratio and non-alcoholic fatty liver disease: the abund study. Rev Assoc Med Bras (1992).

[5] Zeng T, Yu J, Tan L, Wu Y, Tian Y, Wu Q (2018). Noninvasive indices for monitoring disease course in Chinese patients with autoimmune hepatitis. Clin Chim Acta.

[6] Kocaoglu S, Alatli T (2022). The Efficiency of the HALP score and the modified HALP score in predicting mortality in patients with acute heart failure presenting to the emergency department. J Coll Physicians Surg Pak.

[7] Akbas A, Koyuncu S, Hacım NA, Dasiran MF, Kasap ZA, Okan I (2022). Can HALP (hemoglobin, albumin, lymphocytes, and platelets) score differentiate between malignant and benign causes of acute mechanic intestinal obstruction?. Cancer Biother Radiopharm.

[8] Tian M, Li Y, Wang X, Tian X, Pei LL, Wang X (2021). The hemoglobin, albumin, lymphocyte, and platelet (HALP) score is associated with poor outcome of acute ischemic stroke. Front Neurol.

[9] Sargin ZG, Dusunceli I (2022). The effect of HALP score on the prognosis of gastric adenocarcinoma. J Coll Physicians Surg Pak.

[10] Richardson N, Wootton GE, Bozward AG, Oo YH (2022). Challenges and opportunities in achieving effective regulatory T cell therapy in autoimmune liver disease. Semin Immunopathol.

[11] Takahashi A, Ohira H, Abe K, Zeniya M, Abe M, Arinaga-Hino T (2021). Differences in autoimmune hepatitis based on inflammation localization. Med Mol Morphol.

[12] Longhi MS, Mieli-Vergani G, Vergani D (2021). Regulatory T cells in autoimmune hepatitis: an updated overview. J Autoimmun.

[13] Wei Y, Li Y, Yan L, Sun C, Miao Q, Wang Q (2020). Alterations of gut microbiome in autoimmune hepatitis. Gut.

[14] Tsai CY, Hsieh SC, Liu CW, Lu CS, Wu CH, Liao HT (2021). Cross-talk among polymorphonuclear neutrophils, immune, and non-immune cells via released cytokines, granule proteins, microvesicles, and neutrophil extracellular trap formation: a novel concept of biology and pathobiology for neutrophils. Int J Mol Sci.

[15] Xia D, Chen D, Cai T, Zhu L, Lin Y, Yu S (2022). Nimbolide attenuated the inflammation in the liver of autoimmune hepatitis's mice through regulation of HDAC3. Toxicol Appl Pharmacol.

[16] Zhang D, Zeng H, Pan Y, Zhao Y, Wang X, Chen J (2022). Liver tumor markers, HALP score, and NLR: simple, cost-effective, easily accessible indexes for predicting prognosis in ICC patients after surgery. J Pers Med.

[17] Feng JF, Wang L, Yang X (2021). The preoperative hemoglobin, albumin, lymphocyte and platelet (HALP) score is a useful predictor in patients with resectable esophageal squamous cell carcinoma. Bosn J Basic Med Sci.

[18] Xu M, Chen L, Hu Y, Wu J, Wu Z, Yang S (2023). The HALP (hemoglobin, albumin, lymphocyte, and platelet) score is associated with early-onset post-stroke cognitive impairment. Neurol Sci.

[19] Han H, Hu S, Du J (2023). Predictive value of the hemoglobin-albumin-lymphocyte-platelet (HALP) index for ICU mortality in patients with acute exacerbations of chronic obstructive pulmonary disease (AECOPD). Intern Emerg Med.

[20] Tanaka A (2020). Autoimmune hepatitis: 2019 update. Gut Liver.

[21] Wang H, Wang J, Huang R, Xia J, Zuo L, Yan X (2019). Red blood cell distribution width for predicting significant liver inflammation in patients with autoimmune hepatitis. Eur J Gastroenterol Hepatol.

[22] Domerecka W, Homa-Mlak I, Mlak R, Michalak A, Wilińska A, Kowalska-Kępczyńska A (2022). Indicator of inflammation and NETosis-low-density granulocytes as a biomarker of autoimmune hepatitis. J Clin Med.

[23] Kosekli MA (2022). Mean platelet volume and platelet to lymphocyte count ratio are associated with hepatitis B-related liver fibrosis. Eur J Gastroenterol Hepatol.

